# Reconsidering ECG‐Defined Atrial Cardiomyopathy in Population Studies of Stroke Mortality

**DOI:** 10.1111/anec.70164

**Published:** 2026-02-09

**Authors:** Mucahit Yetim, Macit Kalçık

**Affiliations:** ^1^ Department of Cardiology, Faculty of Medicine Hitit University Corum Turkey

To the Editor,

We read with interest the article by Xia et al. examining the joint association of metabolic syndrome (MetS) and electrocardiographically defined atrial cardiomyopathy (AtCM) with stroke mortality in the general population (Xia et al. 2026). Using NHANES III data and deep terminal negativity of the P wave in V1 (DTNPV1) as a surrogate of AtCM, the authors report the highest stroke mortality risk among individuals with concomitant MetS and AtCM. The topic is clinically relevant and addresses an important gap regarding non‐atrial fibrillation substrates of thromboembolism. However, several methodological and interpretative issues merit further consideration.

A major concern relates to the operational definition of AtCM based solely on DTNPV1. Contemporary consensus statements emphasize that atrial cardiomyopathy is a multidimensional construct incorporating electrical, structural, and functional abnormalities, ideally assessed through a multimodal approach including imaging and biomarkers (Goette et al. 2017). Reliance on a single ECG parameter may lead to misclassification and attenuate effect estimates, particularly in population‐based cohorts where atrial remodeling is heterogeneous. Although sensitivity analyses with composite ECG markers were performed, the absence of imaging or biomarker validation limits the specificity of the AtCM phenotype.

The analytic sample selection may also affect external validity. Exclusion of participants without fasting samples or ECGs resulted in the removal of a substantial proportion of the original NHANES III cohort, yielding an older and more metabolically burdened population. While appropriate weighting preserves representativeness of the fasting subsample, the findings may not be generalizable to healthier or younger individuals. Prior population studies have shown that the prevalence and prognostic impact of metabolic risk factors vary considerably across age strata and cardiometabolic profiles (Mottillo et al. 2010), which should temper extrapolation of the reported hazard ratios to the broader population.

Another important limitation is the absence of longitudinal atrial fibrillation (AF) ascertainment. AtCM is increasingly recognized as a substrate predisposing to AF and thromboembolism, yet AF itself remains a strong mediator of stroke risk. Without baseline or incident AF data, residual confounding or mediation cannot be excluded. Although adjustment for P‐wave indices partly addresses atrial electrical remodeling, these markers cannot fully substitute for rhythm surveillance. Prior work suggests that subclinical AF and atrial disease frequently coexist and jointly influence stroke outcomes (Kamel et al. 2015).

Finally, the clinical implications regarding anticoagulation warrant caution. While the authors suggest that individuals with both MetS and AtCM may benefit from preventive strategies, current evidence does not support anticoagulation in the absence of documented AF or established thromboembolic indications. Ongoing randomized trials targeting atrial cardiopathy populations underscore the uncertainty in this area (Kamel et al. 2019). Prospective studies integrating comprehensive AtCM phenotyping, systematic AF detection, and adjudicated ischemic stroke outcomes are required before therapeutic recommendations can be justified.

## Author Contributions

All of the authors contributed planning, writing, and revision.

## Funding

The authors have nothing to report.

## Conflicts of Interest

The authors declare no conflicts of interest.

## Data Availability

Data sharing not applicable to this article as no datasets were generated or analyzed during the current study.
